# Clinical features and surgical treatment of mediastinal masses in children: a retrospective study of 51 cases

**DOI:** 10.3389/fped.2025.1608867

**Published:** 2025-08-20

**Authors:** Xianhui Shang, Yuanmei Liu, Zhen Luo, Yingbo Li, Guangxu Zhou, Hongyang Tan, Kaiyi Mao

**Affiliations:** ^1^ Department of Pediatric Surgery, Affiliated Hospital of Zunyi Medical University, Zunyi, China; ^2^ Department of Pediatric Surgery, Guizhou Children’s Hospital, Zunyi, China

**Keywords:** children, mediastinal mass, imaging examination, pathological type, surgical treatment, bronchial compression, ventilation management, perioperative safety

## Abstract

**Objective:**

To investigate the clinical features, imaging manifestations, pathological types, and surgical strategies of mediastinal masses in children with this condition, aiming to enhance early diagnosis and perioperative management.

**Methods:**

Clinical data of children diagnosed with mediastinal masses and treated at the Affiliated Hospital of Zunyi Medical University between January 2019 and August 2024 were retrospectively reviewed. Key variables analyzed included demographic characteristics, clinical presentation, imaging findings, surgical procedures, intraoperative management, pathological results, and follow-up outcomes. Risk stratification and intraoperative safety strategies were assessed through representative complex case analyses.

**Results:**

A total of 51 children were enrolled, comprising 29 males and 22 females, with a median age of 5.0 years. Primary clinical presentations included shortness of breath upon exertion (54.9%) and cough (49.0%), while 5 children were asymptomatic. All diagnoses were confirmed by computed tomography (CT), with lesions predominantly located in the middle and posterior mediastinum. Ganglioneuroma, bronchogenic cyst, and schwannoma were the most common types. Benign lesions accounted for 84.3%, whereas 8 cases were malignant. Thoracoscopic surgery was performed in 45 children and open thoracotomy in 6. Intraoperatively, iodine solution was applied to cystic lesions in 16 cases, and sclerosing agent injections were administered to 4 lymphangioma cases. Postoperatively, 10 children with malignant tumors required subsequent oncological treatment. One child encountered mechanical ventilation failure during anesthetic induction, which was resolved by transitioning to a prone position. In bronchogenic cysts, preemptive decompression effectively prevented complications such as bronchial obstruction.

**Conclusion:**

Mediastinal masses in children are predominantly benign, frequently presenting with nonspecific respiratory symptoms, and CT remains the diagnostic modality of choice. Surgical resection was the primary treatment. Individualized intraoperative management based on lesion type, appropriate patient positioning, and decompression procedures can significantly reduce complication risks. Preoperative airway compression assessment and collaboration with anesthesia teams to establish emergency ventilation protocols are essential to ensuring perioperative safety in children.

## 1 Introduction

Although mediastinal masses are relatively rare in children, their occurrence can rapidly lead to compression of critical structures such as the heart, lungs, great vessels, and airways due to the limited space and complex anatomy of the mediastinum, potentially causing severe and even life-threatening complications. Unlike adults, mediastinal tumors in children are distinct, typically arising from embryonic remnants, neurogenic proliferation, or cystic lesions, resulting in notable differences in clinical presentation, pathology, and therapeutic strategies. Previous studies have demonstrated that neurogenic tumors, cystic lesions, and lymphatic tissue hyperplasia are the most common mediastinal masses in children; however, systematic analyses regarding their precise incidence, anatomical distribution, and surgical risks remain insufficient.

Clinically, mediastinal masses in children often present with nonspecific symptoms, frequently identified during routine physical examination. Larger masses may cause compressive symptoms, including cough, dyspnea, hoarseness, and dysphagia (1, 2). Chest radiography serves as an initial screening method, typically revealing soft tissue masses obscuring normal anatomical borders (1). Computed tomography (CT) remains the preferred diagnostic modality, providing detailed information regarding lesion location, size, internal characteristics, and the relationship to surrounding anatomical structures (2). Magnetic resonance imaging (MRI) offers supplementary value, especially for evaluating neurogenic tumors and complex lesions due to superior soft-tissue resolution. Ultrasound may also assist in lesion localization and cystic characterization, particularly in younger children or when radiation exposure was a concern. Comprehensive analysis integrating imaging characteristics, demographic features, and laboratory findings enhances diagnostic accuracy (2–4).

Surgery continues to be the cornerstone of treatment for children with mediastinal masses (5, 6). In recent years, minimally invasive thoracoscopic surgery has become mainstream due to advancements in pediatric surgical techniques. However, it is critical to acknowledge that surgical risks in children significantly exceed those in adults, particularly in lesions causing tracheobronchial compression, where anesthetic induction may precipitate acute airway obstruction. This scenario necessitates meticulous preoperative evaluation and prompt intraoperative interventions. Airway compression exceeding 50% on CT imaging has been associated with a significantly increased risk of intraoperative ventilation failure (7). Additionally, bronchogenic cysts require special attention since direct intraoperative traction or dissection may cause cyst rupture, resulting in severe airway obstruction. This observation has been reported in previous clinicopathological studies and must be factored into perioperative planning (8). Thus, comprehensive preoperative assessment and intraoperative vigilance are imperative.

Furthermore, current evidence on mediastinal masses in children was predominantly derived from single-center case series with limited long-term follow-up data. This restricts the generalizability of conclusions regarding recurrence risk and functional outcomes. To address these knowledge gaps, we conducted a single-center retrospective study analyzing clinical data from 51 children diagnosed with mediastinal masses and treated at the Affiliated Hospital of Zunyi Medical University between January 2019 and August 2024. We systematically review clinical characteristics, imaging findings, pathological profiles, and surgical strategies, supplemented by detailed analyses of representative high-risk cases. Comparative insights are also offered between benign and malignant tumors and between thoracoscopic and open surgical approaches. Particular emphasis was placed on preoperative airway evaluation, optimization of intraoperative procedures, and emergency perioperative ventilation management. Our aim was to provide clinicians with comprehensive guidance to improve early recognition, diagnostic precision, and therapeutic safety for children with mediastinal masses.

## 2 Methods

### 2.1 Study design and ethical approval

This was a single-center, retrospective observational study aiming to systematically analyze the clinical characteristics, imaging features, pathological profiles, and surgical management of children diagnosed with mediastinal masses. Participants were children who underwent surgical treatment for mediastinal masses at the Department of Pediatric Surgery, Affiliated Hospital of Zunyi Medical University, from January 2019 to August 2024.

The study protocol was approved by the Ethics Committee of the Affiliated Hospital of Zunyi Medical University (Approval Number: KLL-2024-706), and strictly adhered to the principles outlined in the Declaration of Helsinki. Written informed consent was obtained from all legal guardians prior to surgery.

### 2.2 Inclusion and exclusion criteria

Inclusion criteria: 1. Children aged 0–14 years; 2. Confirmed preoperative imaging diagnosis of mediastinal mass; 3. Surgical treatment performed at our hospital with complete pathological diagnosis; 4. Comprehensive clinical records available, including symptoms, physical examination findings, imaging data, surgical records, and follow-up outcomes.

Exclusion criteria: 1. Non-primary mediastinal origin of lesions; 2. Conservative treatment without surgery; 3. Incomplete imaging or pathological data; 4. Lack of definite postoperative pathological diagnosis.

### 2.3 Data collection

**Figure 1:**
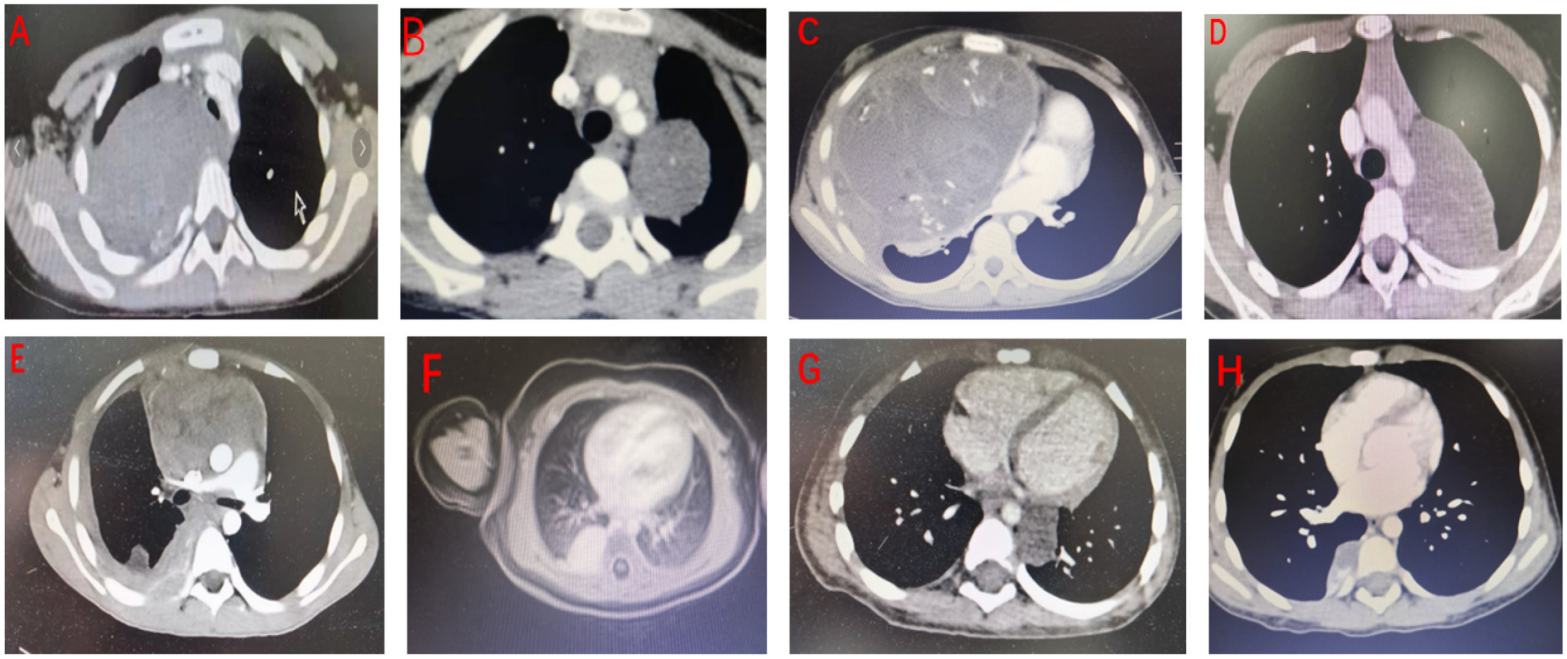
Selected CT images of mediastinal masses from representative patients. Postoperative pathological diagnoses are as follows: **(A)** ganglioneuroma; **(B)** ganglioneuroma; **(C)** mixed germ cell tumor (yolk sac tumor and mature teratoma); **(D)** schwannoma; **(E)** lymphoma; **(F)** hemangioma; **(G)** esophageal duplication cyst; **(H)** extralobar pulmonary sequestration.

Data were retrospectively extracted from the hospital's electronic medical records and surgical databases. Collected information included basic demographic data (gender, age), initial clinical symptoms and physical signs, imaging modalities used and their findings (x-ray, CT, MRI, ultrasound), anatomical location of masses, surgical techniques employed, special intraoperative interventions (e.g., sclerosing agent injection, iodine-based cyst wall treatment, cyst fluid decompression via aspiration), pathological results, postoperative management (including transfer to oncology departments), and follow-up outcomes. Data were independently collected and cross-verified by two investigators, with challenging cases reviewed by a third-party clinical expert. Selected CT images of children with mediastinal masses are shown below (Figure 1).

### 2.4 Imaging and pathological classification

Mediastinal compartments were categorized into anterior, middle, and posterior regions based on standardized radiological anatomical landmarks. All imaging data were independently reviewed by two senior radiologists (associate chief physician or higher). Disagreements were adjudicated by a third radiologist. Final pathological diagnoses were made by two experienced pathologists according to the latest World Health Organization (WHO) tumor classification system and categorized as benign or malignant.

### 2.5 Preoperative evaluation and perioperative management

All children underwent preoperative contrast-enhanced CT to assess the location, size, and extent of the mediastinal mass, as well as its relationship to adjacent structures including the trachea, bronchi, heart, and major vessels. In cases where CT imaging revealed airway narrowing, the degree of tracheobronchial compression was semi-quantitatively assessed and categorized as mild (<25%), moderate (25%–50%), or severe (>50%).

For children with moderate to severe airway compression, a multidisciplinary team comprising pediatric surgeons, anesthesiologists, and thoracic specialists jointly developed an individualized perioperative plan. Key elements included:

1. Preserving spontaneous ventilation during anesthetic induction whenever feasible;
2. Availability of advanced airway equipment such as fiberoptic bronchoscopes and video laryngoscopes;
3. Adjustments in intraoperative positioning to relieve airway compression;
4. Implementation of pre-formulated emergency ventilation protocols.

One child experienced acute ventilation failure during induction, attributed to >60% tracheal compression. Emergency oral-to-endotracheal tube insufflation temporarily restored oxygenation, followed by intraoperative repositioning to the prone position, which restored airway patency and enabled safe surgical continuation. This case highlights the importance of incorporating preoperative airway evaluation and individualized intraoperative strategies in high-risk children.

**Figure 2:**
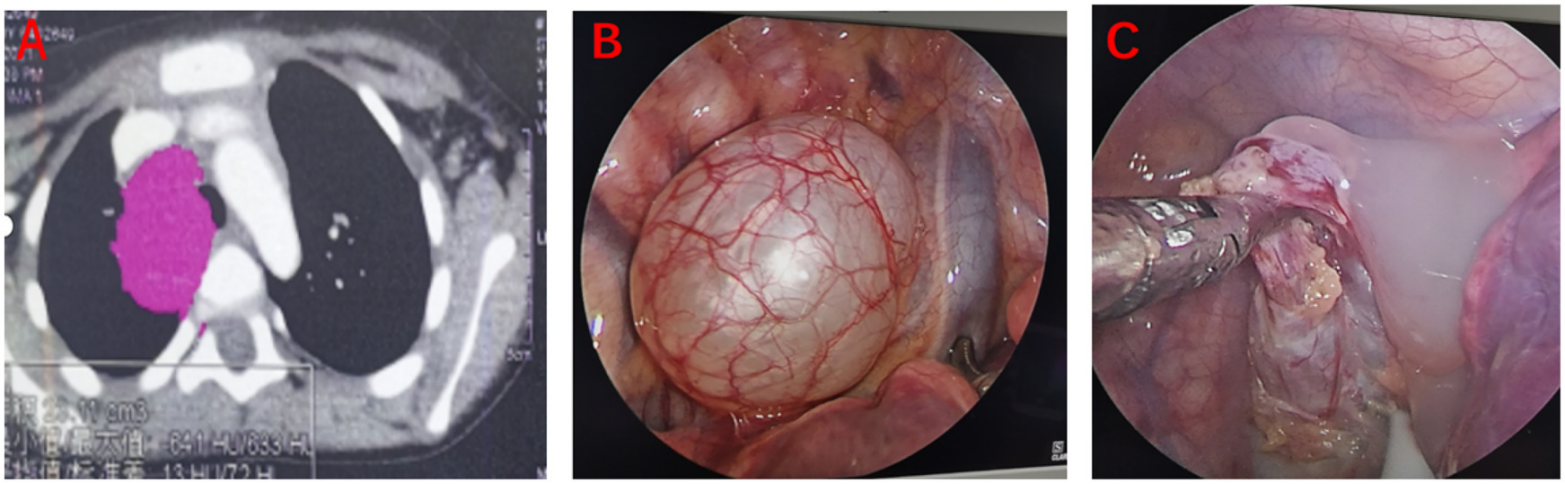
Bronchogenic cyst. **(A)** Preoperative CT scan; **(B)** Intraoperative view of the intact cyst; **(C)** Cystic fluid after decompression.

Particular caution was exercised for bronchogenic cysts, given their frequent anatomical proximity to airway structures. Excessive tension on the cyst wall during dissection posed a high risk of rupture and subsequent obstruction (9). As a preventative measure, all bronchogenic cysts underwent controlled aspiration decompression using an 18G needle under direct vision prior to resection (Figure 2).

For selected cystic lesions (*n* = 16), the residual cyst wall was gently irrigated intraoperatively with 4% tincture of iodine, followed by thorough rinsing with normal saline. This was performed to ablate the secretory function of the cyst epithelium and thereby reduce the risk of recurrence. The technique was adapted from the method reported by Feng et al. (10), in which the inner surface of the cyst was cauterized using 4% iodine tincture, demonstrating favorable efficacy in minimizing postoperative recurrence.

### 2.6 Statistical analysis

All data were entered into Microsoft Excel 2021 and independently cross-verified. Descriptive statistical analyses were performed using SPSS version 29.0. Categorical variables are presented as frequencies and percentages, while continuous variables are reported as mean ± standard deviation or as medians with interquartile ranges, depending on their distribution.

Given the retrospective and descriptive nature of the study, no inferential statistical tests or multivariable modeling were applied. However, subgroup comparisons—such as between children with benign vs. malignant tumors, or between those who underwent thoracoscopic vs. open surgical approaches—were conducted descriptively to enhance clinical interpretability. All results are summarized using appropriate tables and figures.

## 3 Results

### 3.1 Patient demographics and hospitalization characteristics

A total of 51 children diagnosed with mediastinal masses and surgically treated at the Affiliated Hospital of Zunyi Medical University between January 2019 and August 2024 were included in this study. All children had histopathologically confirmed diagnoses. The cohort consisted of 29 males and 22 females (male-to-female ratio, 1.3:1), with a median age of 5.0 years (range: 0.5–12 years). Most cases were managed by the Departments of Pediatric Surgery and Thoracic Surgery, with an average hospitalization duration of 8.3 days (Table 1).

**Table 1:** Demographic distribution and hospitalization duration among children with mediastinal masses.

Variable	Value
Total number of children	51
Male	29
Female	22
Age range (years)	0.5–12
Median age (years)	5.0
Primary departments	Pediatric Surgery
Average hospitalization duration (days)	8.3

### 3.2 Clinical presentations

The most common presenting symptoms were exertional dyspnea (54.9%) and chronic cough (49.0%), followed by sputum production, fever, and chest pain. Notably, 9.8% of children were asymptomatic, with lesions detected incidentally during routine physical examinations (Table 2).

**Table 2:** Clinical symptoms and presenting signs in children with mediastinal tumors.

Clinical symptom	Number of cases	Percentage (%)
Shortness of breath upon exertion	28	54.9
Cough	25	49.0
Sputum production	14	27.5
Fever	6	11.8
Chest pain	5	9.8
Hoarseness	3	5.9
Dysphagia	2	3.9
Asymptomatic	5	9.8

### 3.3 Pathological subtypes and anatomical distribution

Histopathologically, benign lesions comprised the majority (84.3%, 43/51). The most common diagnosis was ganglioneuroma (11 cases), predominantly located in the posterior mediastinum. Eight cases (15.7%) were malignant, including neuroblastomas, lymphomas, and germ cell tumors, with a predilection for the anterior mediastinum (Table 3).

**Table 3:** Histopathological classification and anatomical localization of mediastinal tumors.

Pathological type	Cases	Primary anatomical region	Nature
Ganglioneuroma	11	Posterior mediastinum	Benign
Schwannoma	4	Posterior mediastinum	Benign
Neuroblastoma	3	Posterior mediastinum	Malignant
Germ cell tumor	2	Anterior mediastinum	Malignant
Mixed germ cell tumor	1	Anterior mediastinum	Malignant
Mature teratoma	2	Anterior mediastinum	Benign
Bronchogenic cyst	7	Middle mediastinum	Benign
Esophageal cyst	5	Middle mediastinum	Benign
Other cysts	4	Middle mediastinum	Benign
Lymphoma	4	Anterior mediastinum	Malignant
Lymphangioma	4	Middle mediastinum	Benign
Hemangioma	2	Middle mediastinum	Benign
Extralobar pulmonary sequestration	2	Posterior mediastinum	Benign

### 3.4 Imaging and surgical management

All children underwent CT imaging, which served as the principal diagnostic tool. Chest x-rays initially identified mediastinal abnormalities in 19 cases, while MRI was employed in 12 children to assess tumor invasiveness, and ultrasound guided localization or aspiration in 8. Minimally invasive thoracoscopic surgery was performed in 88.2% (45/51), while 6 children required open thoracotomy due to tumor size, complex anatomical location, or suspected malignancy (Table 4).

**Table 4:** Imaging modalities and intraoperative management strategies.

Procedure/management	Number of cases
Chest x-ray identifying mediastinal shadow	19
Confirmatory CT scan	51
MRI further evaluation	12
Ultrasound-guided localization	8
Thoracoscopic resection	45
Open thoracotomy	6
Intraoperative sclerotherapy (lymphangioma)	4
Intraoperative iodine treatment for cysts	16
Bronchogenic cyst decompression	4
Postoperative pathological examination	51
Postoperative oncology referral	10
Follow-up for benign lesions	41

### 3.5 Intraoperative risk management

Among children with malignant tumors, thoracoscopic resection was feasible in 5 out of 8 cases (62.5%), whereas thoracoscopy was applied in 95.1% (39/41) of those with benign tumors. Malignant tumors were associated with greater intraoperative complexity and longer hospitalization (median stay 10.1 vs. 7.4 days).

Airway compression severity was graded based on CT. Nine children exhibited moderate-to-severe narrowing (compression >25%). One child with 63% tracheal compression experienced acute airway collapse during anesthesia induction. Emergency oral-to-endotracheal tube insufflation, followed by repositioning to the prone position, restored ventilation and permitted completion of surgery without conversion.

### 3.6 Cyst-specific surgical techniques

Bronchogenic cysts presented a high risk of rupture. All such cases underwent intraoperative decompression via fluid aspiration. Additionally, iodine-based cyst wall irrigation was performed in 16 cases, and sclerotherapy was used in 4 lymphangiomas to reduce recurrence risk.

### 3.7 Follow-up outcomes

Follow-up data were available for all children with benign lesions. Follow-up duration ranged from 3–36 months (median: 17 months). No recurrences or functional complications were observed. All 10 children with malignant tumors were referred to oncology for postoperative adjuvant therapy.

### 3.8 Conclusions

This study demonstrates that mediastinal masses in children are predominantly benign and typically present in preschool-aged individuals, with a slight male predominance. Respiratory symptoms, particularly dyspnea on exertion and chronic cough, are the most common presenting features.

CT remains the gold standard for diagnosis and surgical planning, while MRI and ultrasound provide valuable adjunctive information in selected cases. Minimally invasive thoracoscopic surgery was safe and effective for the majority of children, though open thoracotomy remains essential in anatomically complex or malignant lesions.

Airway compression risk stratification using CT was crucial for pre-anesthesia evaluation, and intraoperative airway management, including prone positioning and manual ventilation strategies, should be preemptively prepared. Targeted intraoperative management of cystic lesions, such as decompression and chemical irrigation, can mitigate complication and recurrence risks.

Although long-term outcome data are pending, mid-term follow-up shows excellent prognosis for children with benign lesions, while comprehensive multidisciplinary care remains essential for those with malignant tumors. Prospective multicenter studies are warranted to validate these findings and enhance clinical guidelines (Table 5).

**Table 5:** Summary of clinical conclusions and practice recommendations for pediatric mediastinal tumors.

Key conclusions	Clinical recommendations
Mediastinal masses commonly occur in preschool-aged children, slightly more frequently in boys.	Heightened awareness and screening for mediastinal masses in younger children
Shortness of breath upon exertion and cough are the predominant symptoms.	Early imaging assessment for unexplained respiratory symptoms
CT was the primary diagnostic tool; MRI and ultrasound are complementary.	Contrast-enhanced CT should be first-line to delineate lesion size, location, and compression.
Surgical resection, primarily minimally invasive, was the preferred treatment.	Select individualized surgical approaches based on lesion characteristics, utilizing open surgery when necessary.
High vigilance for bronchial compression; preoperative emergency plans required.	Thorough airway assessment pre-anesthesia; prepare prone positioning and manual ventilation strategies intraoperatively.
Specific intraoperative management of cystic lesions reduces complications and recurrence risk.	Decompression before resection in bronchogenic cysts; postoperative iodine or sclerotherapy as necessary.

## 4 Discussion

Mediastinal tumors in children, occurring within the central thoracic cavity, present a unique clinical challenge due to their propensity to compress vital mediastinal structures such as the trachea, heart, esophagus, and great vessels (11, 12). While open thoracotomy has historically been the standard approach, the advent of minimally invasive thoracoscopic techniques has significantly broadened surgical options for this population (13). The mediastinum's embryologic complexity, derived from all three germ layers, gives rise to a wide variety of tumor types, most of which are benign (14).

Mediastinal lesions are anatomically classified into anterior, middle, and posterior compartments. Anterior tumors typically include thymic, germ cell, and lymphoid neoplasms; middle compartment lesions are often congenital cysts or vascular anomalies; and posterior tumors predominantly comprise neurogenic neoplasms (15–17). Bronchogenic cysts and esophageal duplication cysts, while relatively uncommon, originate from abnormal foregut budding and account for 13%–15% of congenital thoracic lesions (18).

In children, surgical management must account for narrower airways, limited cardiopulmonary reserve, and anatomical variability. These features necessitate a refined approach to preoperative risk assessment and intraoperative planning. Our findings align with established imaging-based compartmental frameworks (4, 7), emphasizing the importance of vigilant airway evaluation, particularly in anterior and middle mediastinal tumors.

### 4.1 Tumor distribution and age-dependent histopathology

In our cohort, benign tumors comprised 84.3% of cases, with ganglioneuromas (21.6%) and bronchogenic cysts (13.7%) most prevalent. These lesions were mainly located in the posterior and middle mediastinum. Malignant lesions, including lymphomas and neuroblastomas, represented 15.7% of cases and were predominantly anterior. This compartment-specific distribution reflects developmental origins and was consistent with prior pediatric series (1).

Among posterior mediastinal neurogenic tumors, schwannomas were characterized by slow growth and encapsulation (19), while neurofibromas occurred either as isolated lesions or in association with neurofibromatosis type 1 (20, 21). Ganglioneuromas, derived from sympathetic ganglia, remained the most common and least aggressive.

### 4.2 Imaging modalities are essential due to nonspecific symptoms

Most children presented with nonspecific symptoms such as exertional dyspnea or chronic cough, which often mimicked upper respiratory conditions. In 9.8% of cases, tumors were detected incidentally during routine evaluations, underscoring the importance of thorough screening and diagnostic imaging.

Computed tomography (CT) achieved 100% diagnostic sensitivity in this study and remains the primary tool for defining tumor location, morphology, and relationship to adjacent structures. Magnetic resonance imaging (MRI) added value in evaluating paraspinal extension or vascular involvement. Ultrasonography was also beneficial, particularly in younger children, offering radiation-free guidance during cyst aspiration or localization.

### 4.3 Surgical treatment was effective with individualized intraoperative strategies

All children underwent complete surgical resection, with thoracoscopic approaches employed in 88.2% of cases. Minimally invasive surgery was feasible in 95.1% of benign cases, but only 62.5% of malignant cases, likely due to size, invasion, or anatomical constraints. Children undergoing thoracotomy had a longer median hospitalization (10.1 vs. 7.4 days), indicating greater perioperative burden.

Intraoperative strategies were adapted to tumor characteristics. In the management of cystic lesions, we employed an intraoperative strategy involving irrigation of the residual cyst wall with 4% tincture of iodine. This approach aimed to chemically ablate the secretory function of the cyst epithelium and thereby reduce the risk of postoperative recurrence. The irrigation was performed gently, followed by thorough rinsing with normal saline, which enhanced the local therapeutic effect while minimizing chemical irritation to surrounding tissues. Among the 16 children who received this treatment, no recurrence was observed during the follow-up period. These findings suggest that this intraoperative adjunctive technique was both feasible and safe, and may be worthy of further application in similar cases (10). For lymphangiomas, intraoperative sclerotherapy further minimized recurrence risk.

Bronchogenic cysts posed a notable airway hazard. To prevent rupture-induced airway compromise, we performed direct-vision decompression using an 18G needle prior to dissection, a strategy consistent with Limaïem et al. (9). This approach proved effective in avoiding intraoperative airway emergencies.

### 4.4 High vigilance for airway compression was essential

In one case, tracheal compression >60% led to acute ventilation failure upon anesthetic induction. Emergency manual ventilation via oral endotracheal insufflation, followed by repositioning the child into the prone position, immediately restored airway patency and allowed safe surgical continuation.

CT imaging enabled semi-quantitative grading of airway narrowing: mild (<25%), moderate (25%–50%), or severe (>50%). This grading informed anesthetic strategy. The critical case with 63% compression supports previous findings that compression beyond 50% markedly increases intraoperative respiratory risk (2).

For high-risk children, we coordinated multidisciplinary planning, preserved spontaneous respiration during induction, ensured advanced airway equipment availability, and rehearsed emergency repositioning protocols. These precautions were instrumental in preventing catastrophic outcomes.

### 4.5 Strengths and limitations

This study was among the few to integrate anatomical location, imaging severity, pathological subtype, and intraoperative technique into a structured perioperative framework for mediastinal tumors in children. The inclusion of real-world high-risk cases reinforced the clinical value of individualized approaches.

Nonetheless, limitations include its retrospective single-center design and limited sample size, which may introduce selection bias. Long-term data remain incomplete; however, mid-term follow-up (3–36 months) of 41 children with benign tumors showed no recurrence or functional sequelae. Five-year outcome tracking is ongoing. Additionally, lack of multivariate analysis limits causal interpretation.

Future prospective, multicenter studies are needed to confirm our findings and further optimize perioperative management protocols.

## 5 Conclusion

Mediastinal tumors in children are primarily benign but can pose significant perioperative risks, especially when associated with airway compression. CT remains the diagnostic cornerstone, guiding surgical and anesthetic planning. Tailored intraoperative strategies—such as cyst decompression, chemical sclerotherapy, and dynamic positioning—can enhance safety and effectiveness. A multidisciplinary, anatomy-driven approach was essential in achieving optimal outcomes in our pediatric mediastinal surgery cases.

## Data Availability

The original contributions presented in the study are included in the article/Supplementary Material, further inquiries can be directed to the corresponding author.
